# State of the AI: Post-Deployment Monitoring of Radiology-Focused Internally Developed AI

**DOI:** 10.1016/j.mcpdig.2026.100342

**Published:** 2026-02-01

**Authors:** Cole J. Cook, Jason R. Klug, Blaize W. Kandler, Abraham Baez-Suarez, Adam P. Dachowicz, Daniel J. Blezek, Andrew D. Missert, Gian Marco Conte, Justin D. Benfield, Amanda Mensing-Diggs, Matthew T. Edwards, Michele A. Powell, Emily N. Sheedy, Holly M. Meyer, Joseph Melnick, Bryce F. Flor, David E. Vidal, Vera Sorin, B. Selnur Erdal, Steve G. Langer, Jeremy D. Collins, Eric E. Williamson, Panagiotis Korfiatis, Timothy L. Kline

**Affiliations:** a Department of Radiology, Mayo Clinic, Rochester, MN; b Information Technology, Mayo Clinic, Rochester, MN; c Center for Digital Health, Mayo Clinic, Rochester, MN; d Department of Radiology, Mayo Clinic, Jacksonville, FL

## Abstract

Articles on the development of medical image artificial intelligence (AI) algorithms are numerous in the literature, but deployment to clinical practice is infrequently discussed. The Enterprise Radiology Framework for AI Software Technology Team at Mayo Clinic has been focused on bridging the gap in clinical translation of medical image AI algorithms since its inception in 2019. During this time, we have released 17 algorithms into our radiology clinical practice. Recently, we have placed an increased focus on monitoring these algorithms, as there are few reports with practical experience documented in the literature. Our increased monitoring efforts include daily, weekly, and yearly monitoring of utilization, failure modes, data drift, and end-user feedback through automated alerts, dedicated dashboards, and pointed investigations to enable optimal algorithmic processing. End-user feedback is elicited yearly during annual reviews to ensure clinical needs are still being met. Automated monitoring has enabled earlier identification of problems, such as images no longer routing through the orchestration engine to the appropriate algorithm, minimizing potential disruption to the clinical practice and ensuring continued algorithmic utilization. Monitoring has also reinforced the importance of key aspects of interdisciplinary research and translation, such as early discussions on clinical needs coupled with technological ability and proper training. By providing our experience in and continuing to improve monitoring methods as a community, we can all minimize risk and maximize the benefits of medical pixel-based AI.

## Graphical abstract

**Figure:**
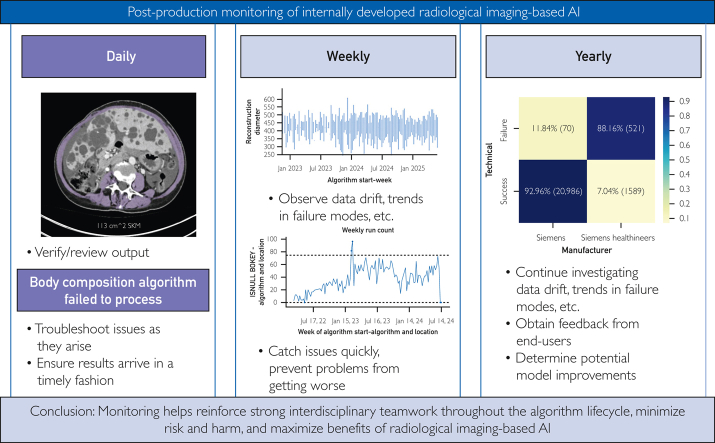

Artificial Intelligence (AI) is increasingly being integrated into radiology. Although AI can enhance diagnostic accuracy and streamline workflows,^1^^,^^2^ integration of these tools presents challenges.^3^^,^^4^ These include the importance of consistent and reliable performance and clinical relevance in practice. Addressing these challenges requires post-deployment monitoring to ensure algorithms continue to perform as expected.^5^^,^^6^

Adequate monitoring requires considering any changes that may impact algorithm performance, such as data drift, concept drift, a relational change between input and output variables, and a change in clinical context.^7^ Numerous articles exist describing methods for detecting data drift in health care in general^8^^,^^9^ and in radiology specifically.^10^^,^^11^ Examples of radiology-specific data drift include changes in image acquisition parameters and protocols, new scanners, new pulse sequences in magnetic resonance imaging, and new detector technologies.

Recommendations exist for developing and implementing an AI monitoring system sensitive to performance-modifying changes in health care.^6^^,^^12^^,^^13^ Though some examples exist for monitoring AI in health care,^13^ few, if any, articles exist describing practical experience in monitoring radiology algorithms.^14^^,^^15^ This paper aims to fill this gap by describing the post-deployment monitoring methods and results for internally developed algorithms in a radiology clinical practice by the Enterprise Radiology Framework for AI Software Technology (FAST) Team at Mayo Clinic. In addition, we discuss how monitoring reinforces strong interdisciplinary teamwork, ensuring these applications remain clinically relevant and adequate in the radiologists’ workflow. (see graphical abstract)

## Methods

### Algorithm Deployment

Before deployment, Digital Imaging and Communications in Medicine (DICOM) tag-based routing rules are defined for our orchestration engine (DEWEY).^16^ DEWEY is a workflow orchestrator that listens for new DICOM studies and classifies them by tags. It launches tag-specific workflows (often dedicated containers) whose tasks include submission of algorithm executions (through simple linux utility for resource management)^17^ to high performance compute clusters. DEWEY passes the image locations and parameters to each job, monitors completion, and then wraps AI outputs into DICOM objects (eg, DICOM structured report objects, DICOM segmentation, etc.) that are routed back to Picture Archival and Communication System or other systems.

Algorithm deployment begins with a multiphase limited rollout and development of a post-deployment monitoring plan. The initial limited rollout phase verifies that the previously defined DICOM tag-based routing rules and computational resource management, such as a number of graphics processing units, meet the desired clinical specifications. These include ensuring routing rules include required imaging series and processing times are sufficient. A prospective reader-study in the clinical workflow is then performed with radiologist volunteers to ensure the algorithm performs as expected and meets clinical need.^3^ If this reports a safe and efficacious AI with risks and hazards minimized, the algorithm is fully rolled out, becoming generally available to all users, typically first at a single site and then potentially to other sites. As the algorithm becomes available to more sites and users, the DICOM tag-based routing rules are refined to ensure proper series routing.

Currently, the FAST Team has rolled out 17 internally developed, medical image-based algorithms, averaging over 10,000 algorithm runs weekly. Following the nomenclature of Rezazade et al,^18^ these algorithms aid in processing (n=5) and perception (n=12) tasks (Table). Clinical implementation considerations, performance, and workflow enhancements have been presented for specific algorithms previously.^23^ However, most of our algorithms are clinical, not workflow improvements, meaning the main benefits are related to providing richer information, which may not be provided otherwise. Thus, limited information is available regarding clinical workflow effects.

**Table:** Information on the 17 Algorithms Currently in Clinical Practice From Mayo Clinic’s Enterprise Radiology FAST Team

Algorithm	Type	Full Rollout	Modality	Location	Average Weekly Run Count
AI-based generalizable algorithm for multi-energy (AGATE)^19^	Image processing	November 16, 2023	CT	MCR, MCF	3
AI denoising for CT	Image processing	September 21, 2023	CT	MCR	132
Aorta (3D laboratory)	Segmentation	December 9, 2021	CT	MCR, MCA	395
Automated liver and spleen volumes in CT	Segmentation/quantification	October 3, 2024	CT	MCR	451
Automatic renal characterization—tumor identification and classification (ARCTIC)^20^	Segmentation	April 18, 2024 (Limited)	CT	MCR	59
AutoFuse	Image processing	December 7, 2024	PET/CT or PET/MR	MCR	648
Body composition^21^	Segmentation/quantification	January 28, 2021	CT	MCR, MCA, MCF, MCHS	8158
Carotid image rendering using projections (CIRUP)	Segmentation	June 27, 2024	CT	MCR	4
Circle of Willis (COW) carotid—3D laboratory	Segmentation	July 1, 2021	CT	MCR	159
Glenoid bone—3D Laboratory	Segmentation	December 9, 2021	CT	MCR, MCHS	13
MR angiography automated projection for long-term efficiency (MAAPLE)	Segmentation	September 1, 2022	MR	MCR	48
Mayo automated tractography algorithm (MATA)	Image processing	May 12, 2022	MR	MCR	29
Prostate segmentation for radiation oncology (organ at risk/OAR)	Segmentation	April 10, 2021	CT	MCR, MCA	32
Quantitative stone analysis software (QSAS-AI)	Segmentation/quantification	July 13, 2023	CT	MCR, MCF, MCA, MCHS	98
Renal donor—3D laboratory	Segmentation	April 16, 2024	CT	MCR, MCF	180
Simultaneous chemical exchange saturation transfer overlay on anatomic images (SCONE)	Image processing	March 20, 2025	MR	MCR	4
Total kidney/liver volumes (TKLV) for PKD/PLD^22^	Segmentation/quantification	November 1, 2019 (TKV) September 16, 2021 (TKLV)	MR, CT	MCR, MCF, MCA, MCHS	16

Abbreviations: CT, computed tomography; FAST, Framework for AI Software Technology; MCA, Mayo Clinic Arizona; MCF, Mayo Clinic Florida; MCHS, Mayo Clinic Health Systems; MCR, Mayo Clinic Rochester; MR, magnetic resonance; PET, positron emission tomography.

### Human-in-the-Loop Integration

Our algorithms all have a human-in-the-loop component, a key guideline developed for good machine learning in medicine.^24^ Four segmentation algorithms undergo initial review by advanced imaging specialists trained in segmentation correction before clinician inspection and subsequent 3D rendering or printing for patient education or surgical planning.

**Figure 1:**
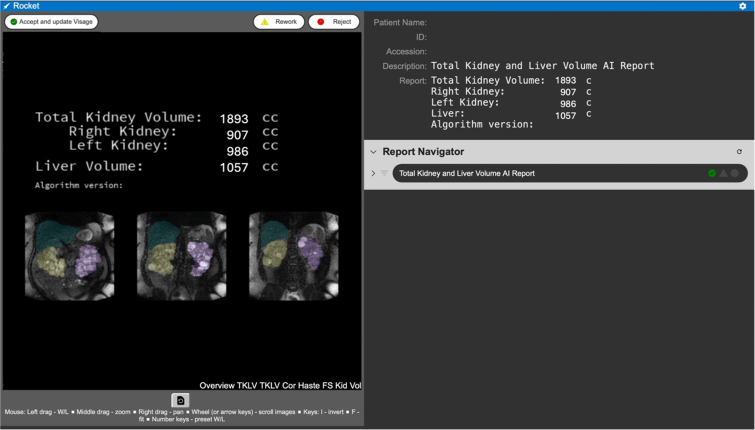
Demonstration of ROCKET interface for TKLV, an algorithm which provides volumes of the kidneys and liver for individuals with polycystic kidney/liver disease. Radiologists can accept the segmentation and corresponding volumes by clicking the accept and update Visage button in the top left corner with the green circle inclosing a white checkmark. They can choose to send the series to image analysts if modifications to the segmentation are needed by clicking the rework button with the yellow caution sign. Finally, the radiologists can reject the segmentation by clicking the reject button with the red circle. A ROCKET reject typically occurs when a series is too noisy for metrics to be calculated. On the right, the report navigator allows radiologists to view the output from other algorithms which may have been run on series from this study. Note the algorithm output values have been modified. TKLV, total kidney and liver volumes.

Four other algorithms are reviewed by radiologists through ROCKET, an in-house image viewer^25^ (Figure 1). Three ROCKET interactions are available: accept, rework, or reject. Accepted outputs are routed to a Picture Archival and Communication System, such as Visage (Visage Imaging), and their corresponding metrics can be edited and integrated into radiology reports at the radiologist’s discretion through macros in PowerScribe 360 (PS360; Nuance Communications, Inc), a speech recognition tool for creating radiology reports. Results are attributed as AI-generated. Advanced imaging specialists correct reworked series using clinical post-processing tools such as TeraRecon (TeraRecon, Inc). Noisy series are often rejected.

The remaining algorithms are reviewed by appropriate personnel, but no formal interactions are captured.

Individuals reviewing output are encouraged to submit a ServiceNow (ServiceNow Inc) ticket when errors are found. These tickets are automatically triaged by severity and investigated for root cause. Tickets are routed to information technology (IT) team members who gather insight on the user or observer of the issue, compare observed against expected behavior, and ensure efficacious mitigations. Fixes may be planned for future enhancement. The individual who originally submitted the ticket is notified when the issue has been resolved.

### Post-Deployment Monitoring Framework

Though FAST was initially created in 2019 to develop and deploy algorithms to clinical practice, our monitoring efforts began in earnest in the Spring of 2024 through the creation of algorithm-specific post-deployment monitoring plans, including performance objective surveillance and risk and hazard analysis.^12^ Post-deployment monitoring in clinical environments informs the accuracy of probability estimates of issues or events that may result in harm and the effectiveness of mitigating controls.

**Figure 2:**
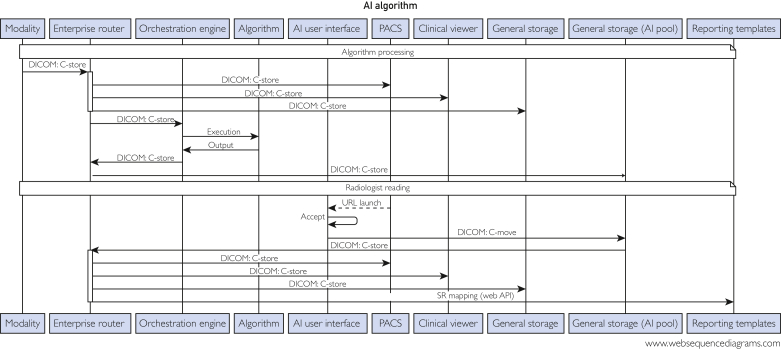
Example sequence diagram of image routing for one of our algorithms, QSAS-AI. After patient imaging, the images are routed to numerous sources. The enterprise router uses the DICOM header information to determine which, if any, algorithms should be run on each series. The enterprise router tags images, allowing the orchestration engine to run the appropriate algorithm (Algorithm). Images can be viewed in either a PACS viewer or our in-house clinical viewer. Source images are stored in a general storage for images and the AI-generated results are specifically stored in the AI pool of the general storage, which has restricted access. Finally, results may be routed to reporting templates to facilitate ease of inclusion in the patient’s radiology report. DICOM, digital imaging and communications in medicine; PACS, picture archiving and communication system; QSAS-AI, quantitative stone analysis software – artificial intelligence.

Though monitoring occurs during all deployment phases, monitoring after full rollout is essential to ensure the safety and efficacy of medical AI. Following full rollout, monitoring occurs at numerous timescales and encompasses multiple data sources. Each system and interaction in Figure 2, an exemplar algorithm sequence diagram, is monitored, either by our team or another, to ensure proper functioning. We will focus on monitoring of our orchestration engine,^16^ the algorithm processing, and ROCKET interactions.^25^

These 3 components allow us to monitor an important metric: algorithmic utilization. This is tracked in several algorithm-specific ways. The first, and most general, is the number of algorithm runs. A series may be processed by an algorithm due to predefined routing rules even if the outputs are not utilized by an end-user. This minimizes wait times for algorithmic results but overestimates true utilization.

The second method for tracking utilization for four algorithms is through ROCKET interactions. Though likely the most accurate method, ROCKET interactions may underestimate utilization because radiologists can view and use algorithm results without providing an official ROCKET designation.

The third method for 2 algorithms is tracking the number of algorithm orders. Unfortunately, an algorithm order does not imply utilization. For these algorithms to run, the appropriate imaging protocol, which includes the required imaging series, must be selected by the protocoling radiologist. If an algorithm is ordered but the necessary protocol is not selected, the algorithm will not trigger, making the number of algorithm orders an overestimate of utilization. Conversely, if a protocoling radiologist selects an appropriate protocol when the algorithm was not ordered, the algorithm will still trigger, making the number of algorithm orders an underestimate of utilization.

The final method of tracking utilization is if the algorithm or its results are mentioned in the radiology report. For algorithms with templated output, templates are often modified in a radiologist-dependent manner, impeding tracking usage. Unfortunately, not all the algorithms produce easily quantifiable metrics. Fused or denoised images, for example, often are used without mention. Therefore, radiology reports containing algorithmic results may not reflect all utilization.

Outside of utilization, we also capture data regarding technical and algorithmic failures as part of an ongoing medical algorithm audit.^26^ Examples of technical failures are a compute node being out of memory or missing a required package. Algorithmic failures are technical successes because the code successfully completed and output was produced, but the output may be not diagnostically helpful, such as an incorrectly placed region of interest. Monitoring algorithmic failures requires a human-in-the-loop, often reported through ServiceNow tickets. For 3 segmentation algorithms, final human-corrected masks elucidate algorithmic failures, allowing root cause analysis and curating a dataset for future model development.

Finally, image acquisition parameters and patient demographics help investigate data drift and failure mode root cause analyses.

### Monitoring Frequency and Statistical Methods

Monitoring is performed at multiple timescales. Daily monitoring, using both automated and human-in-the-loop components, ensures algorithms are running smoothly and output is available as needed. The automated component is done using Splunk (a real-time monitoring and troubleshooting solution; Splunk LLC). Email alerts are sent to appropriate IT staff for events such as technical failures or high memory usage, enabling a high degree of technical fidelity. Human-in-the-loop components are mainly through ServiceNow tickets.

**Figure 3:**
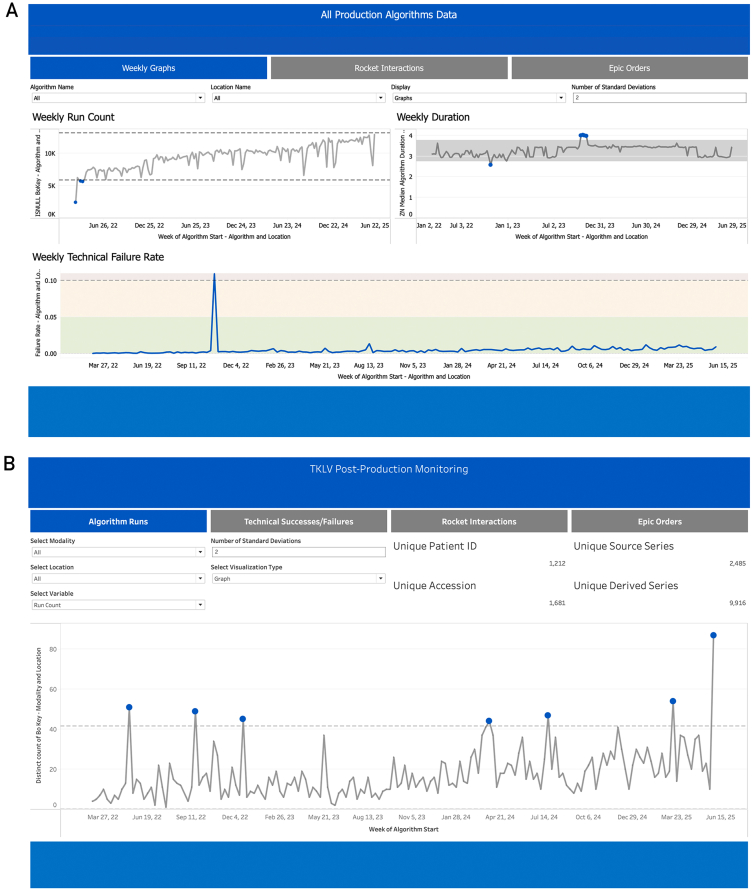
Examples of Tableau dashboards used for algorithmic monitoring: (A) the omnibus dashboard allows investigation into the run counts, durations, and technical failure rates for all algorithms together or each algorithm individually while (B) a dashboard for an individual algorithm allows for an in-depth investigation.

Weekly monitoring identifies trends that are hard to detect from daily monitoring while enabling action before adverse effects to the clinical practice worsen. Utilization, failures, and data drift are monitored weekly by Tableau (Salesforce, Inc) dashboards (Figure 3) and Jupyter^27^, ^28^, ^29^ notebooks/Python (Python Software Foundation, https://www.python.org)^30^ scripts. The dashboards provide data to leadership (see Figure 3A for program level investigation) and individuals outside the FAST Team, such as the original algorithm proponent (see Figure 3B for an individual algorithm example). FAST team members rely on Jupyter notebooks/Python scripts to investigate potential performance issues. Graphical methods utilized include histograms, violin plots, and statistical process control charts. Control limits for count data, such as utilization metrics, are based on counts of data prior to the current calendar year to account for temporal changes in a total number of scans. Control limits for image acquisition parameters and patient demographics are based on developmental data. These values may update as adequate performance is reported with new image acquisition parameters or patient demographics. Various color-coding is employed, especially between technical successes and failures, as an initial root cause analysis along with appropriate statistical tests/metrics such as the Kolmogorov-Smirnov test, χ^2^ test, and Jensen-Shannon divergence. Alerts happen when new values of categorical variables, such as new scanner models, occur and when continuous variables have values outside previously observed ranges.

Finally, all data, including end-user feedback, are collated during algorithm-specific annual reviews. These reviews are required by our steering committee, the Enterprise Radiology Artificial Intelligence Subcommittee. During these reviews, we explore what may impact performance, such as comparing distributions of image acquisition parameters leading to technical successes and failures using Kolmogorov-Smirnov, Mann-Whitney U, χ^2^, or other appropriate statistical tests. For algorithms with corrected masks saved out, relations between the Dice coefficient and image acquisition parameters are investigated graphically and via regression models. Along with insights gained from failure mode investigations, end-user feedback guides potential algorithmic enhancements. This feedback also elucidates the sentiments of our algorithms in practice—an extremely valuable intangible metric of success. Finally, annual components of post-deployment plans are reviewed.

## Results

Monitoring results are categorized by the main signal, leading to further investigation starting with measures of algorithm utilization, specifically weekly run counts and ROCKET interactions. Then, we discuss failure modes, specifically technical failures where no output is produced. Finally, we demonstrate how solicited feedback drives innovation.

### Weekly Run Counts

**Figure 4:**
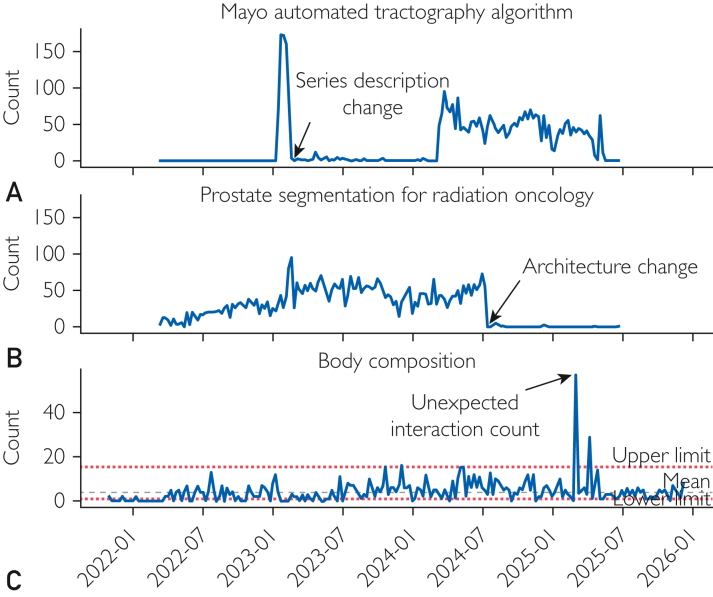
Plots of the (A) weekly algorithm run counts for the Mayo automated tractography algorithm, (B) weekly algorithm run counts for the prostate segmentation for radiation oncology algorithm, and (C) the weekly ROCKET interaction counts for the body composition algorithm. Weekly algorithm run counts dropped to zero for (A) the Mayo automated tractography algorithm due to changes in series description and (B) the prostate segmentation for radiation oncology algorithms due to changes in technical architecture. Both changes caused appropriate series to no longer route to either algorithm. The weekly ROCKET interaction counts (C) for the body composition algorithm are plotted using a statistical process control chart with mean value in the dashed gray line and upper and lower limits in dotted red lines. The large peak in (C), labeled as unexpected interaction count, corresponded to a new user interacting with cases for which the body composition algorithm results were not needed while interacting with results from a different algorithm.

Weekly run counts provide a quick check that images are routed appropriately. For two algorithms, weekly run counts dropped to zero (Figure 4A, B). We noticed this both by a weekly email listing algorithm run counts for the previous week and individual statistical process control charts. Root cause analysis began similarly for both: investigate image series routing from the scanner.

For our tractography algorithm (Figure 4A), no series matching the expected series descriptions were routed. We reached out to the MR technologists and determined the series description was changed, preventing appropriate routing. While routing rules were updated, the tractography algorithm was run as requested by radiology IT support staff. The routing rules were eventually modified to automatically route appropriate series without intervention again.

In the case of our prostate segmentation algorithm, series matching the expected series description were still being acquired (Figure 4B). Discussions with the proponent suggested recent technical architecture changes resulted in appropriate routing no longer being possible. However, they suggested an alternative model was available and currently deployed for other situations. Formal model decommissioning followed, freeing up technical infrastructure and reducing potential confusion between functionally similar models.

### ROCKET Interactions

Of our utilization metrics, ROCKET interactions are likely the most directly related to clinical utilization. While investigating information from the previous week, we noted ROCKET interactions for our body composition algorithm were much higher than expected (Figure 4C). Multiple interactions occurred from a single new user for both the body composition algorithm and the quantitative stone analysis software algorithm. This user was interested in the quantitative stone analysis software results but mistakenly interacted with the body composition results due to the ROCKET interface listing. We clarified only results of interest should be interacted with as ROCKET interactions allow information to be included in the patient record. This led to refinement of our educational materials.

### Failures Modes

**Figure 5:**
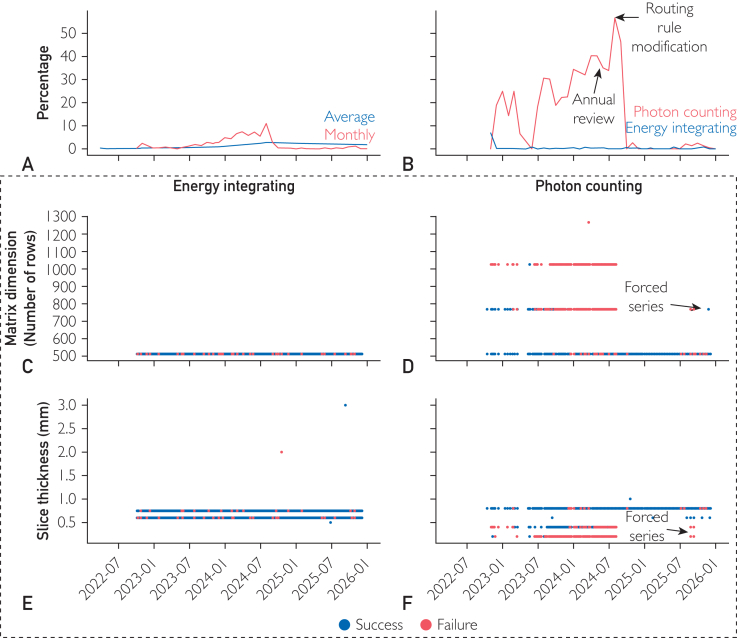
A collection of graphs which reports the root cause analysis for determining why numerous technical failures were occurring. The monthly technical failure percentage in red continued to rise through much of 2024 while the running average in blue remained within a reasonably low percentage of less than 5% (A). The 2024 annual review verified the high technical failure rate of photon counting CT scanners compared to the energy integrating CT scanners (B). The 2024 annual review further revealed it was not the photon counting CT scanners themselves but the large matrix dimensions (C and D) and thin slices (E and F) that lead to technical failures (red points). The routing rules were modified (B) to reduce the number of scans, which would likely lead to technical failures though a few series which did not meet the routing rules were forced to the algorithm (D and F). CT, computed tomography.

Failure modes often require accumulating examples to resolve. For instance, one of our models, a segmentation model for the Circle of Willis and carotid arteries, frequently encountered technical failures (Figure 5A). These failures were often documented in ServiceNow tickets by the advanced image specialists who review and modify the segmentation prior to 3D rendering. When technical failures occurred, these advanced image specialists worked the segmentation from the ground up, which increased the median turnaround time from 19 minutes when an AI-generated segmentation was available to 44 minutes without.

As individual tickets arrived, root cause analysis was difficult as each case appeared unique and underlying patterns were hard to observe. Thus, we further investigated root cause during the 2024 algorithm-specific annual review. Many of the technical failures observed were from the new photon counting detector computed tomography (CT) scanners (Figure 5B; χ^2^ test statistic=2799.70, DOF=1, *P*<.001). However, not all photon counting CT series created technical failures. Additional investigation suggested these technical failures were due to large image matrices and thin slices often obtained with these new scanners (Figure 5C-F). The DICOM-tag-based routing rules were modified to exclude images larger than 512 × 512 or with slices less than 0.5 mm, whereas a new model was developed that would address any contrast and image size differences between energy integrating and photon counting CT scanners.

These routing rule modifications reduced the overall technical burden and prevented processing delays because the large images often processed for over 60 minutes before ultimately terminating. These long processing times often exceeded the median turnaround time for the advanced image specialists to produce an unaided segmentation. Modifying the routing rules therefore not only reduced the overall technical burden but also prevented further delaying the advance image specialists and subsequently, the reading radiologists and clinicians who utilize the 3D renderings in their clinical work.

### End-User Feedback

Feedback is solicited from all algorithm end-users prior to annual review. During the 2024 annual review for AutoFuse, an algorithm which registers a PET series with the appropriate CT or MR series and determines the radiotracer standardized uptake value, we were told this algorithm was extremely useful and a huge timesaver due to the number of cases processed daily. The most frequently mentioned problem was that it did not work on more radiotracers. A formal enhancement request was completed and the project was prioritized. Without this feedback, we would not have known the next steps for this highly successful and well utilized algorithm.

## Discussion

Post-deployment monitoring is essential for algorithms in clinical practice to minimize the risk of patient or operator harm and maximize benefit, such as significant time savings, decreased cognitive load, and/or improved patient care. Our monitoring efforts align with many of those suggested by multiple radiological societies around the world,^5^ such as monitoring for data drift in imaging acquisition parameters, which may influence model performance. Increasing our monitoring efforts has reinforced the importance of strong interdisciplinary teamwork, such as inclusion of appropriate stakeholders, communication strategies, training, and ensuring quality care^31^^,^^32^ throughout the algorithmic cycle to maximize utilization.

Monitoring algorithmic utilization, though basic, provides a wealth of information and insight. Before implementing widespread monitoring in 2024, radiologists often notified us of missing algorithm results, delaying their report finalization and potential treatment planning by downstream clinicians. With a more formal monitoring process, we can now proactively detect and address these problems to maximize utilization and minimize user dissatisfaction and impact to clinical workflows. As DICOM tag modification prevents proper routing, inclusion of appropriate stakeholders who may modify DICOM tags, such as scanner technologists and medical physicists, is necessary to ensure images continue to be routed appropriately.

Ensuring compatibility between clinical need and technical parameters is paramount not only in the early stages of development but also during clinical utilization. For instance, the Circle of Willis Carotid algorithm was initially developed only using data from energy integrating CT scanners and the corresponding image acquisition parameters used in the clinic at the time. However, requisite series from these new CT scanners frequently led to technical failures due to large image sizes. Communications with appropriate stakeholders ensured routing rule modifications to exclude large images would minimally affect their clinical workflow and confirmed model improvements to address differences in image contrast and dimensions between energy integrating and photon counting CT scanners were needed. Open communication ensured frustrations were minimized and cordial relations continued.

Solicited feedback has provided possible modifications that would improve the algorithm in clinical practice and reported the added value of these tools. Neither of these would have been known without feedback. This highlights the crucial importance of continued communication when developing and deploying AI to ensure the AI remains relevant to clinical practice.

## Conclusion

Since its inception in 2019, our team has helped deploy numerous internally developed algorithms to clinical practice. Beginning in earnest in 2024, we have seen numerous benefits from the implementation and increased emphasis of monitoring AI deployed in clinical practice. We hope our experience can help guide others and that through continued interdisciplinary teamwork and methodological improvements throughout the algorithmic cycle, we can all minimize the risk of harm and maximize the clinical benefits of integrating AI tools into radiology practice.

## Potential Competing Interests

The authors report no competing interests.
